# Studying *Coxiella burnetii* Type IV Substrates in the Yeast *Saccharomyces cerevisiae*: Focus on Subcellular Localization and Protein Aggregation

**DOI:** 10.1371/journal.pone.0148032

**Published:** 2016-01-28

**Authors:** María Rodríguez-Escudero, Víctor J. Cid, María Molina, Jan Schulze-Luehrmann, Anja Lührmann, Isabel Rodríguez-Escudero

**Affiliations:** 1 Dpto. de Microbiología II, Facultad de Farmacia, Universidad Complutense de Madrid and Instituto Ramón y Cajal de Investigaciones Sanitarias (IRyCIS), Madrid, Spain; 2 Mikrobiologisches Institut-Klinische Mikrobiologie, Immunologie und Hygiene, Universitätsklinikum Erlangen, Friedrich-Alexander Universität Erlangen-Nürnberg, Erlangen, Germany; Texas A&M Health Science Center, UNITED STATES

## Abstract

*Coxiella burnetii* is a Gram-negative obligate parasitic bacterium that causes the disease Q-fever in humans. To establish its intracellular niche, it utilizes the Icm/Dot type IVB secretion system (T4BSS) to inject protein effectors into the host cell cytoplasm. The host targets of most cognate and candidate T4BSS-translocated effectors remain obscure. We used the yeast *Saccharomyces cerevisiae* as a model to express and study six *C*. *burnetii* effectors, namely AnkA, AnkB, AnkF, CBU0077, CaeA and CaeB, in search for clues about their role in *C*. *burnetii* virulence. When ectopically expressed in HeLa cells, these effectors displayed distinct subcellular localizations. Accordingly, GFP fusions of these proteins produced in yeast also decorated distinct compartments, and most of them altered cell growth. CaeA was ubiquitinated both in yeast and mammalian cells and, in *S*. *cerevisiae*, accumulated at juxtanuclear quality-control compartments (JUNQs) and insoluble protein deposits (IPODs), characteristic of aggregative or misfolded proteins. AnkA, which was not ubiquitinated, accumulated exclusively at the IPOD. CaeA, but not AnkA or the other effectors, caused oxidative damage in yeast. We discuss that CaeA and AnkA behavior in yeast may rather reflect misfolding than recognition of conserved targets in the heterologous system. In contrast, CBU0077 accumulated at vacuolar membranes and abnormal ER extensions, suggesting that it interferes with vesicular traffic, whereas AnkB associated with the yeast nucleolus. Both effectors shared common localization features in HeLa and yeast cells. Our results support the idea that *C*. *burnetii* T4BSS effectors manipulate multiple host cell targets, which can be conserved in higher and lower eukaryotic cells. However, the behavior of CaeA and AnkA prompt us to conclude that heterologous protein aggregation and proteostatic stress can be a limitation to be considered when using the yeast model to assess the function of bacterial effectors.

## Introduction

*Coxiella burnetii*, a Gram-negative obligate intracellular bacterial species is a widespread zoonotic pathogen which causes the Q-fever syndrome in humans. A variety of wild and domestic birds, fish and mammals act as reservoirs in the epidemiologic cycle of this pathogen [1]. Infection in humans is produced after the inhalation of contaminated aerosols, generated by domestic livestock activities. Symptomatic acute Q-fever manifests as a flu-like illness, usually self-limiting. However, occasionally, chronic Q-fever can develop and evolve into a more serious disease, often involving endocarditis or hepatitis [2].

*C*. *burnetii* expresses a type IVB secretion system (T4BSS) homologous to the Dot/Icm system of phylogenetically related *Legionella pneumophila* [3]. *C*. *burnetii* uses its T4BSS to deliver virulence factors into the host cell cytoplasm, where they interplay with host cell signaling pathways, thus adapting the host physiology to the demands of the infecting pathogen. A major key to virulence lies in the fact that this manipulation occurs within professional phagocytes in the host, such as macrophages, leading to the survival and replication of bacteria in the ‘parasitophorous vacuole’ [4]. This compartment shares features with phagolysosomes, but is inefficient in digesting the bacteria. The identification and functional study of the translocated bacterial effectors involved is an important challenge for the understanding of *C*. *burnetii* biology and the molecular determinants of disease.

*C*. *burnetii* is fastidious to manipulate genetically, although important advances have been made in the latest years [5]. Hence, great part of the knowledge derives from genetic analyses of *Legionella pneumophila* as alternative host to screen for possible Dot/Icm-translocated candidates, supported by bioinformatics to identify putative effectors [6]. Functional studies in alternative eukaryotic model organisms as counterfeit hosts by means of heterologous expression is also a widespread approach to understand the function of bacterial translocated effectors. The budding yeast *Saccharomyces cerevisiae*, in virtue of its ease of manipulation and the impressive amount of knowledge about its biology, has been widely exploited [7]. We have previously used this model to infer functional traits of translocated effectors from *Salmonella enterica* ser. Typhimurium [8–10] and enteropathogenic *E*. *coli* [10]. Other researchers have exploited yeast to study effectors from several pathogens, including obligate intracellular *Chlamydia trachomatis* [11] and, recently, *C*. *burnetii* [12–14]. Thus, the yeast system has stood out as a powerful tool to study bacterial effector proteins. As such proteins target eukaryotic pathways, they often lead to traceable effects on yeast.

Here we report expression of a set of *C*. *burnetii* proteins in *S*. *cerevisiae* as a platform for their study, and discuss its validity. We chose to express three *C*. *burnetii* proteins with ankyrin repeat domains, AnkA, AnkB and AnkF [15, 16] as well as the anti-apoptotic effectors CaeA and CaeB [17] and a novel candidate effector, CBU0077. Although ankyrin repeats are common structural domains in eukaryotic cells, including yeast, the molecular function and interactions of this *C*. *burnetii-*secreted protein set remains obscure. Interestingly, CaeA (CBU1524) and CBU0077 had already been shown to inhibit yeast growth [6, 13], suggesting interference with conserved eukaryotic pathways, although their localization had not been determined. In some cases, like those of AnkA and CaeA, the observed activity in yeast seemed to derive from stress induced by protein aggregation, which might be unrelated to the function of the effector protein in the actual context of infection. The localization of ectopically expressed effectors in protein quality control compartments seems to reveal an important limitation of the yeast model. Nevertheless, effectors of yet unknown function, like AnkB and CBU0077, are shown to localize in yeast and higher cells similarly, which might lay the path to dissect the molecular mechanism of these *C*. *burnetii* proteins.

## Materials and Methods

### Bacterial and yeast strains, media and growth conditions

Bacterial genes were amplified from genomic DNA from *C*. *burnetii* Nine Mile phase II clone 4. *S*. *cerevisiae* strains used were YPH499 (*MATa ade 2–101 trp1-63 leu2-1 ura3-52 his3-200 lys2-801*) and BY4741 (*MATa his3Δ1 leu2Δ0 met15Δ0 ura3Δ0*) for general purposes; and VHY87 (*MATα leu2-3*, *112 ura3-52 his4 can1*^*R*^ *TRP1*::*DsRed-HDEL*), a gift of M. Cyert, (Stanford University, CA, USA) for the visualization of the ER. The whole genome deletion (WGD) collection of *S*. *cerevisiae* in the BY4741 strain (EUROSCARF) was used to pick out particular mutants, like *mca1Δ*::*kanMX4* or functionally-oriented subcollections when necessary. The *E*. *coli* strain DH5α F′(K12Δ(*lacZYA*-*argF*)U169 *deoR supE44 thi-1 recA1 endA1 hsdR17 gyrA96 relA1* (*ϕ80lacZΔM15*)F′) was used for molecular biology techniques.

YPD (1% yeast extract, 2% peptone and 2% glucose) broth or agar was the general nonselective medium used for growing yeast. For plasmid selection and maintenance, synthetic complete medium (SC) contained 0.17% yeast nitrogen base without amino acids, 0.5% ammonium sulfate, and 2% glucose, and was supplemented with appropriate amino acids and nucleic acid bases. SCGal and SCRaf media were SC with 2% galactose or raffinose, respectively, instead of glucose. Induction of genes under the *GAL1* promoter was achieved by growing cells in SCRaf broth to log phase and then adding galactose from a 20% (w/v) stock solution to a final concentration of 2% and incubating for 5–8 h at 28–30°C.

For assessment of growth on solid media, 5 μl of cell suspensions at OD_600_ = 0.5 of fresh transformants from preinocula grown overnight in SC lacking uracil, leucine or tryptophan, as required, were spotted onto SC or SCGal plates, also lacking the appropriate auxotrophic selection markers. The original and three serial 1/10 dilutions were deposited for each clone analyzed and growth was recorded after 2 (glucose) or 3 days (galactose) at 28–30°C (standard conditions) or 37°C (thermal stress).

### Molecular biology techniques and plasmid construction

The pEGFP vectors and pCMV-HA were from Clontech (Mountain View, CA, USA). To clone *ankA*, *ankB*, *ankF*, *CBU0077*, *caeA* and *caeB*, the genes were amplified by PCR from genomic DNA of *C*. *burnetii* NMII strain; *ankA*, *ankF*, *caeA*, *caeB* and *CBU0077* were cloned into pEGFP-C2; *ankB* was cloned into pEGFP-C1. The following primers were used: AnkA-EcoRI-F (5´-ccggatccatggcgtgcaggggaatc-3´), AnkA-BamHI-R (5´-aaggatccttaaaacagtccggggcct-3´), AnkB-PstI-F (5´-ccctgcagtatgtttaaccaattggaaatagt-3´), AnkB-KpnI-R (5´-ccggtaccttacatgtgcttacccggg-3´), AnkF-PstI-F (5´-cccctgcagtatgagacagcgtgaaattaat-3´), AnkF-BamHI-R (5´-aaggatccctaccgctggaagccgc-3´). The CaeA and CaeB constructs [17] and the CBU0077 [6] construct are described elsewhere. Additionally CBU0077 was cloned into pCMV-HA with the primers: CBU0077-BglII-F (5´-ccagatctgcatgagacaactcgtttcaattaa-3´) and CBU0077-KpnI-R (5´-ccggtaccttacataatagaacacccacga-3´). pEGFP-C1-hPKCθ was kindly provided by S. Ghosh (Columbia University, NY, USA). To clone *ankA*, *ankB*, *ankF*, *CBU0077*, *caeA* and *caeB* into the yeast expression pYES2-GFP plasmid, these genes were amplified by PCR from pEGFP-AnkA, -AnkB, -AnkF, -CBU0077, -CaeA and -CaeB plasmids respectively. The primers used for these amplifications were AnkA-UP (5’-ggaattcccttgcagttggcagcccg-3’) and AnkA-LO (5’-gctctagattaaaacagtccggggcc-3’), AnkB-UP (5’-ggaattcccatgtttaaccaattggaaa-3’) and AnkB-LO (5’-gctctagattacatgtgcttacccg-3’), AnkF-UP (5’-ggaattcccatgagacagcgtgaaattaat-3’) and AnkF-LO (5’-gctctagactaccgctggaagccg-3’), CBU0077-UP (5’-ggaattcccatgagacaactcgtttca-3’) and CBU0077-LO (5’-gctctagattacataatagaacaccca-3’), CaeA-UP (5’-cgggatccatgaacacaagtcctacatc-3’) and CaeA-LO (5’-ggaattcttaatgatgatgatgatgatgtgtccttttgggagc-3’), and CaeB-UP (5’-cgggatccttggcaggcatagctg-3’) and CaeB-LO (5’-cgggatccttacttattaaattcgggtatg-3’) respectively. The upper primers used to amplify *ankA*, *ankB*, *ankF*, *CBU0077* and *cae*A had *Eco*RI and the lower primers *Xba*I sites. *caeB* upper and lower primers carried a *Bam*HI restriction site. PCR products were cleaved with *Eco*RI and *Xba*I or *Bam*HI, respectively, to be inserted into the same restriction sites of the pYES2-GFP plasmid [18], generating pYES2-GFP-AnkA, pYES2-GFP-AnkB, pYES2-GFP-AnkF, pYES2-GFP-CBU0077, pYES2-GFP-CaeA and pYES2-GFP-CaeB plasmids. To obtain pEG (KG)-AnkB, expressing GST-AnkB, *ankB* was amplified with the AnkB-KG-UP (5’-gctctagacatgtttaaccaattggaa-3’) and AnkB-LO (as above) primers, both of them containing the *Xba*I restriction site, using pYES2-GFP-AnkB as a template. The PCR product was cleaved with *Xba*I and inserted into the same site in the pEG(KG) vector [19]. The pEG(KGH)-AnkB was obtained by subcloning the *ankB*-containing *Xba*I-*Xba*I fragment from pEG(KG)-AnkB into the same site of the pEG(KGH) plasmid, a pEG(KG) plasmid in which the *URA3* marker has been substituted by the *HIS3* marker [20]. The GAL-*YBH3* overexpression plasmid was obtained from a yeast genomic collection (Dharmacon, GE Healthcare). pGILDA-Bax was a gift of J.L. Revuelta (Univ. Salamanca, Spain). pESC-LEU-CHFP-Ubq9ts and p425-GAL1-RNQ1-mCherry [21] were kindly provided by D. Kaganovich (Hebrew University of Jerusalem, Israel). The NOP1-mCherry construct (pUN100-mCherry-NOP1) [22] was a gift of Olivier Gadal (Université de Toulouse, France). Yeast transformation by the lithium acetate method were performed by standard methods

### Microscopy techniques

For fluorescence microscopy of GFP-expressing live yeast cells, log-phase cultures were harvested by centrifugation, washed once with sterile water and viewed directly. *In vivo* DAPI staining was performed by adding DAPI (Sigma) resuspended in PBS at a final concentration of 10 μg·ml^−1^ to cells and incubating for 5 min. To detect vacuolar and endosomal compartments, staining with FM4-64 was performed, as described by Vida and Emr [23]. For statistical significance of data, ≥100 cells were examined for each condition or experiment and triplicate biological replicas were analyzed. Cells were examined under an Eclipse TE2000U microscope (Nikon, Tokyo, Japan) and digital images were acquired with an Orca C4742-95-12ER charge-coupled-device camera (Hamamatsu Photonics, Hamamatsu City, Japan) and HCImage software. Confocal images were taken using an Olympus FV1200 system (Olympus, Tokyo, Japan).

### Immunodetection in yeast extracts by Western blotting

Standard procedures were used for yeast cell growth, collection, breakage, protein separation by SDS-PAGE, and transfer to nitrocellulose membranes [24]. GFP fusion proteins were detected using monoclonal anti-GFP antibodies (Living Colors, JL-8) diluted 1:2000. GST fusions were immunodetected with primary rabbit polyclonal anti-GST (Z-5) antibody (Santa Cruz Biotechnology) diluted 1:2000. Ubiquitin residues were detected using monoclonal anti-ubiquitin antibody (P4G7; Abcam) diluted 1:2000. Secondary antibodies were IRDye-680 or IRDye -800 anti-rabbit or anti-mouse antibodies (Li-Cor Biosciences), or Alexa-680 anti-mouse (Invitrogen) and detection was performed with an Odyssey Infrared Imaging System (LI-COR Biosciences).

### Evaluation of cell death and oxidative damage by flow cytometry

For the analysis of cellular lysis, oxygen-free radicals (ROS) and variations of the mithocondrial membrane potential, yeast transformants were grown in SCRaf medium lacking uracil at 30°C overnight. Then, the cultures were induced by galactose addition to a 2% final concentration for 16 h. Propidium iodide (0.005%), dihydroethidium (2.5 μg/ml for 5 min) or rhodamine 123 (5 μg/ml for 2h) were added; the samples with propidium iodide were diluted 1:10 in PBS. Three thousand cells were analysed per second on a FACScan flow cytometer (Becton Dickinson) on the FL2 log scale. WinMDI 2.7 software was used to handle the graphics obtained.

### Sensitivity assays

Yeast sensitivity or resistance to different compounds was tested by a halo assay on agar plates. Transformants bearing plasmids expressing each of the *Coxiella* proteins or the control empty vector were grown and OD_600_ adjusted to 1. Then, cultures were swabbed uniformly across a SCGal agar plate. A 6 mm cellulose disk, impregnated with the compound to be tested, was then placed on the surface of the agar and plates were incubated for 3 days at 30°C. The diameters of the inhibition haloes were recorded and compared to the corresponding control halos obtained with yeast transformed with the pYES2-GFP empty vector. The compounds and concentrations used were the following: amphotericin B (30 mM), benomyl (25 mM), geneticin (10 mM), menadione (10 mM), hygromycin B (50 mM), latrunculin B (2.5 mM), myriocin (12.5 mM), miconazol (50 mM), rapamycin (2.5mM), tunicamycin (10 mM), cyclosporine A (50 mM), hydroxyurea (50 mM), orthovanadate (50 mM) and neomycin sulfate (100 and 200 mM).

For the determination of dose-response curves, yeast transformants carrying the *GAL1*-promoter bearing plasmid pYES2GFP (empty vector) or pYES2GFP-AnkB were cultured in the appropriate SCRaf medium until log-phase. These preinocula were then diluted into fresh SCRaf to an OD_600_ of 0.5. Then, 5 μL of the suspension were seeded into 96-well microplates containing 100 μL SCGal to induce the expression of the heterologous proteins. Previously, hygromycin B, neomycin sulfate or rapamycin were added in two-fold serial dilutions to the plates. Plates were then incubated at 30°C and OD_600_ was measured at 0, 24, and 48 h in a spectrophotometer for 96-well plates (model 680; Bio-Rad, Hercules, CA).

### HeLa cells transfection, fluorescence microscopy and immunoblotting

HeLa human epithelial cell lines were cultured at 37°C in 5% CO_2_ in Dulbecco modified Eagle medium (Life Technologies) containing 10% heat-inactivated fetal bovine serum (Biochrom) and 1% penicillin-streptomycin (Life Technologies). Cells were plated on coverslips and were transfected with pEGFP-derived plasmids expressing *C*. *burnetii* genes using Xtreme Gene 9 transfection reagent from Roche. Eighteen hours post-transfection, cells were fixed with 4% paraformaldehyde (Alfa Aeser) in PBS (Biochrom), permeabilized with ice-cold methanol, and quenched with 50mM NH_4_Cl (Roth) in PBS. Samples were incubated with the following primary antibodies: anti-tubulin (Sigma, diluted 1:100), anti-calnexin (Stressgen, 1:100) and anti-LAMP1 H4A3 (Developmental Studies Hybridoma Bank, 1:250); and the following secondary antibodies: Alexa Fluor 488 and Alexa Fluor 594 (Life Technologies, 1:600). Cells were mounted using ProLong Diamond with DAPI (Life Technologies) to visualize the nucleus. For mitochondrial staining, cells were incubated using Mitotracker Red CMXRos (Molecular Probes) before fixation according to the manufacturer protocol. Confocal fluorescence microscopy was performed using a Zeiss LSM 700 confocal microscope.

Ectopic expression of GFP, GFP-AnkA, GFP-AnkB, GFP-CaeA, GFP-CaeB and GFP-CBU77 in transiently transfected HeLa cells was analyzed with rabbit anti-GFP (Life Technologies, 1:3000) and anti-α-tubulin (Sigma, 1:5000) antibodies as loading control. Lysate preparation, SDS-PAGE and transfer to PVDF membranes were performed by standard procedures. Proteins were visualized by using horseradish peroxidase-conjugated secondary antibodies (Dianova, 1:5000) and a chemiluminescence detection system (Thermo Scientific or Millipore).

### Ubiquitination Assays

Ubiquitination assays in yeast cells were performed by pulling down GST or GST-CaeA from yeast lysates as described [8], followed by immunoblotting with monoclonal anti-ubiquitin antibodies (ab24686, Abcam) diluted 1:1000. Ubiquitination assays in mammalian cells were performed as described [25]. Briefly, HEK293T cells were transfected in 6-well plates with 1 μg of the pEGFP-derived plasmids and 2 μg of an HA-ubiquitin expressing vector (Addgene plasmid # 18712; a gift from Edward Yeh [26]) or pcDNA3.1 (Life Technologies) as a negative control, using XtremeGene 9 transfection reagent. Eighteen hours post-transfection, cells were treated for 4 h with 20 μM of the proteasome inhibitor MG132. The positive control (PKCθ) was additionally stimulated with 25 nM PMA 45 min before harvesting the cells in 200 μl boiling SDS lysis buffer (20 mM Tris-HCl, 1% SDS, pH 8.0). After boiling and shearing the DNA by ultrasonication, the protein concentration was normalized and 400 μg of total protein were immunoprecipitated overnight in 1 ml TNT buffer (1% Triton X-100, 150 mM NaCl, 20 mM Tris-HCl, 1 mM EDTA, 0.5% NP-40) using 1 μg of rabbit anti-GFP antibody (Life Technologies) and Protein A/G Plus-Agarose (Santa Cruz Biotechnology). Beads were washed extensively and samples were blotted and probed with rabbit anti-GFP (Life Technologies, 1:3000) and mouse anti-HA (HA.11 Clone 16B12, Covance, 1:1000) as primary antibodies. Chemiluminiscent detection was carried out as above.

## Results

### Ectopic expression of the *C*. *burnetii* effector proteins AnkA, AnkB, AnkF, CaeA, CaeB and CBU0077 in HeLa cells

**Fig 1:**
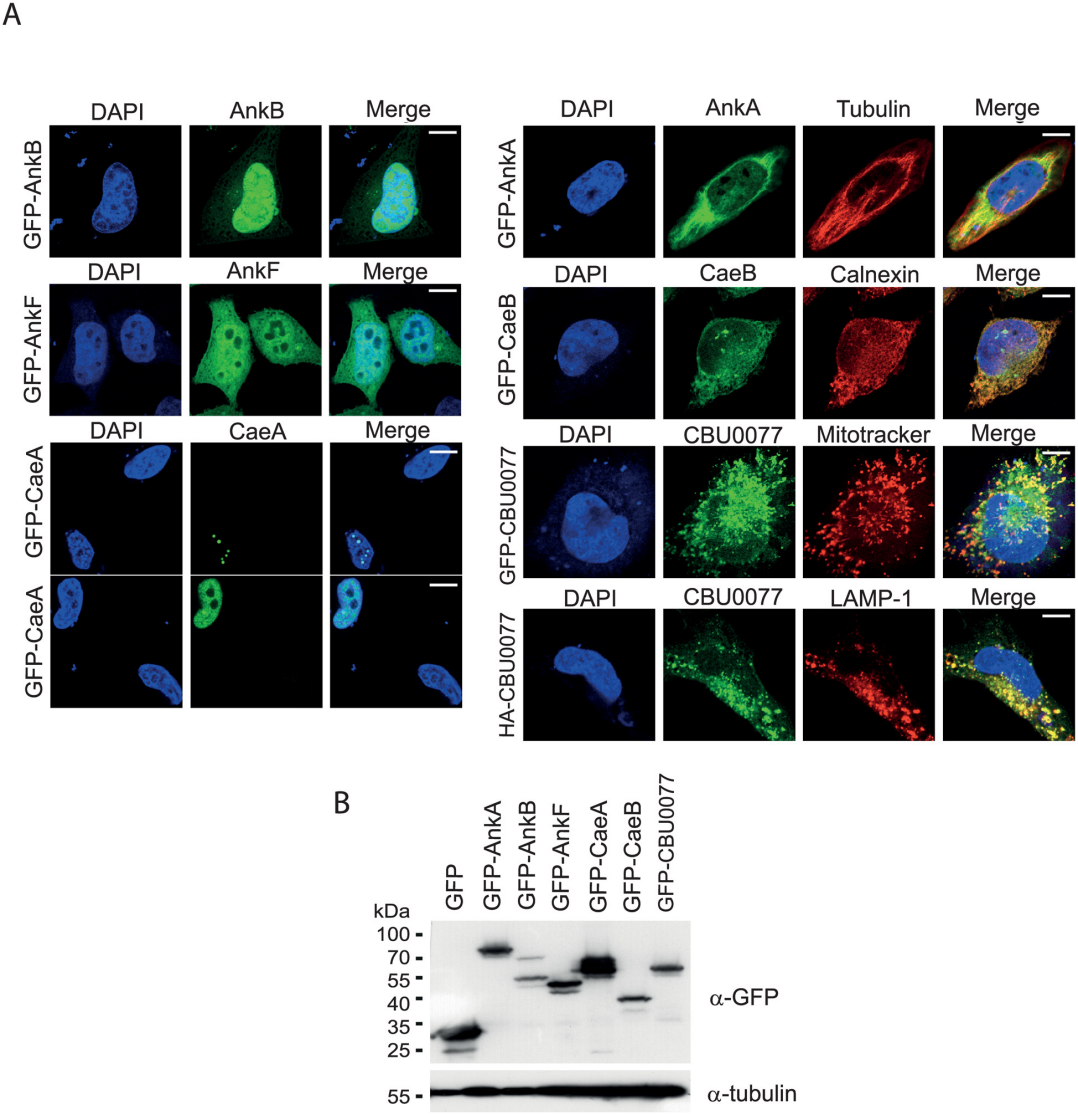
Expression of *C*. *burnetii* T4BSS-translocated effectors in mammalian HeLa cells. (A) Immunofluorescence confocal images show the representative subcellular localization of the indicated GFP- or HA-tagged *C*. *burnetii* effector proteins (green) after ectopic expression in HeLa cells. For contrast, DAPI, Mitotracker, or anti-tubulin, anti-calnexin and anti-LAMP-1 antibodies, were used as marked. Scale bars represent 10mm. (B) Expression of the indicated GFP-tagged *C*. *burnetii* effectors was analyzed by immunoblot analysis. Protein extracts from transfected HeLa cells were separated by SDS-PAGE, transferred to PVDF membrane and probed with anti-GFP antibody and anti-tubulin as loading control. A representative immunoblot is shown out of three independent experiments with similar results.

To evaluate the soundness of *S*. *cerevisiae* as a model to study the putative host cell targets of *C*. *burnetii* T4BSS effectors, we studied in parallel the expression of N-terminal GFP-tagged fusions in both transfected cell lines and yeast. First, we determined their intracellular localization in the human cell line HeLa. As shown in Fig 1A, the different *C*. *burnetii* effector proteins displayed distinct subcellular localization patterns when ectopically expressed. GFP-AnkA partially co-localized with tubulin (see detailed images in S1 Fig), GFP-AnkB displayed nuclear localization, and GFP-AnkF exhibited both cytoplasmic and nuclear localization. Although GFP-CaeA localized diffusely to the nucleus, as reported earlier [6], in roughly 40% of the GFP-CaeA-expressing cells this protein was observed at multiple bright nuclear punctate dots. Finally, CaeB co-localized with the ER marker calnexin. The localization of HA-tagged AnkA, AnkB, AnkF, CaeA and CaeB, was identical to the GFP-tagged versions (data not shown). However, interestingly, the localization of CBU0077 depended on the fusion tag. Thus, GFP-CBU0077 was shown to localize to the mitochondria, while HA-CBU0077 rather co-localized with the lysosomal marker protein LAMP-1 (Fig 1A), in agreement with the previous report that localization of FLAG-CBU0077 was lysosomal [6]. Expression of the GFP-fusion proteins was analyzed using immunoblot analysis (Fig 1B). Notably, GFP-AnkA, -AnkF, -CaeA, -CaeB and -CBU0077 were stably expressed, while GFP-AnkB showed some degradation.

### Expression of *C*. *burnetii* AnkA, AnkB, CBU0077 and CaeA proteins in *S*. *cerevisiae* negatively affects cell growth

**Fig 2:**
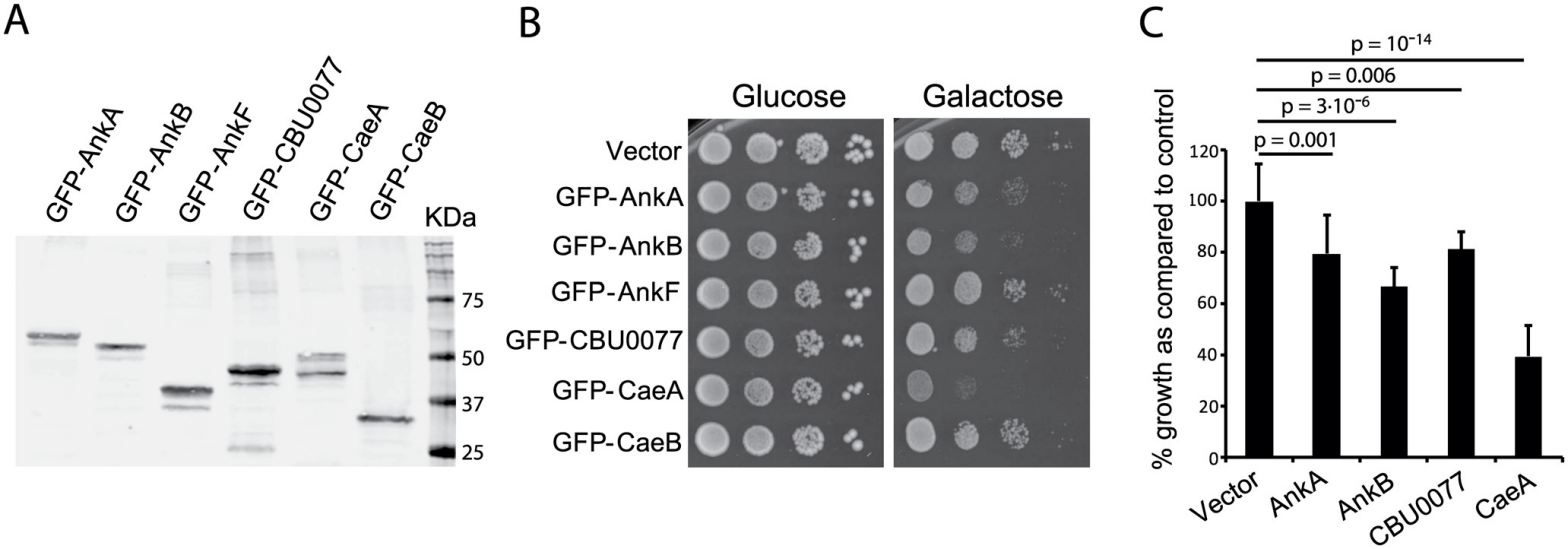
Expression of *C*. *burnetii* T4BSS-translocated effectors in yeast. (A) Immunoblot with anti-GFP antibodies on yeast lysates from transformants of YPH499 strain with pYES2-GFP-derived plasmids expressing the indicated GFP-tagged *C*. *burnetii* effector proteins grown on SCRaf selective medium and induced for 4 h by addition of galactose. (B) Serial dilution drop assays to monitor growth under induction (Galactose) and control (Glucose) conditions of representative transformants as in (A). (C) Growth in liquid SCGal medium of yeast transformants expressing the indicated GFP fusions after 36 h of growth in 96-well plates. Data are the average of 36 wells per experiment, corresponding to three different transformant clones (12 wells per clone), and are expressed in percentage with respect to the control transformed with the empty vector (average O.D._600_ of control samples was considered 100% growth). Bars correspond to the standard deviation. P-values noted were determined by the Student’s T-test.

Next, the ORFs encoding AnkA, AnkB, AnkF, CBU0077, CaeA and CaeB were cloned in frame with GFP in a *GAL1* promoter-driven vector, to be expressed in *S*. *cerevisiae* specifically in the presence of galactose as carbon source. Growth in galactose was assessed, as compared to control conditions in glucose, where the promoter is subjected to catabolic repression. As shown in Fig 2A, all proteins were efficiently expressed in yeast. GFP-AnkA, GFP-AnkB, GFP-CBU0077 and GFP-CaeA led to significant growth inhibition at different degrees, both on solid and liquid media (Fig 2B and 2C), while GFP-AnkF and GFP-CaeB did not (Fig 2B). Moderate growth inhibition of *S*. *cerevisiae* by CBU077 and CaeA is consistent with previous data [6, 13]. The *ankA*, *ankB*, *ankF* and *caeA* genes were additionally expressed as N-terminal GST fusions, leading to the same results (S2 Fig). Given that a large tag may be masking functional domains or result in altered intracellular localization, as shown for ectopic expressed CBU0077 in HeLa cells (Fig 1), some effectors were also expressed as C-terminal tagged fusions to poly-His. Both CBU0077 and CaeA failed to induce toxicity in yeast when expressed as C-terminal poly-His tagged proteins (S2 Fig), in contrast to the N-terminal GFP/GST fusions, implying that toxicity or subcellular targeting of these proteins in yeast depended on their free C-terminal ends.

Incubation of *C*. *burnetii* effector-expressing yeast transformants at 37°C, a suitable temperature for the study of the molecular properties of human pathogens, but a heat-stress temperature for yeast, did not generally alterthe effects on growth caused by the assayed *C*. *burnetii* effectors (S2 Fig). An exception was GFP-AnkB, which was unable to inhibit yeast growth at this temperature. These observations suggested that AnkB is less stable at this temperature in yeast cells. Accordingly, the amount detected by immunoblot on lysates incubated at 37°C was greatly diminished (data not shown). GST-AnkB, however, maintained toxicity (S1 Fig), probably due to the fact that the pEG(KG) vector used imposes a higher expression level that the one used for GFP fusions. A summary of results obtained with the different fusions tested is provided in S1 Table.

### CaeA induces oxidative stress and apoptosis in the yeast cell

**Fig 3:**
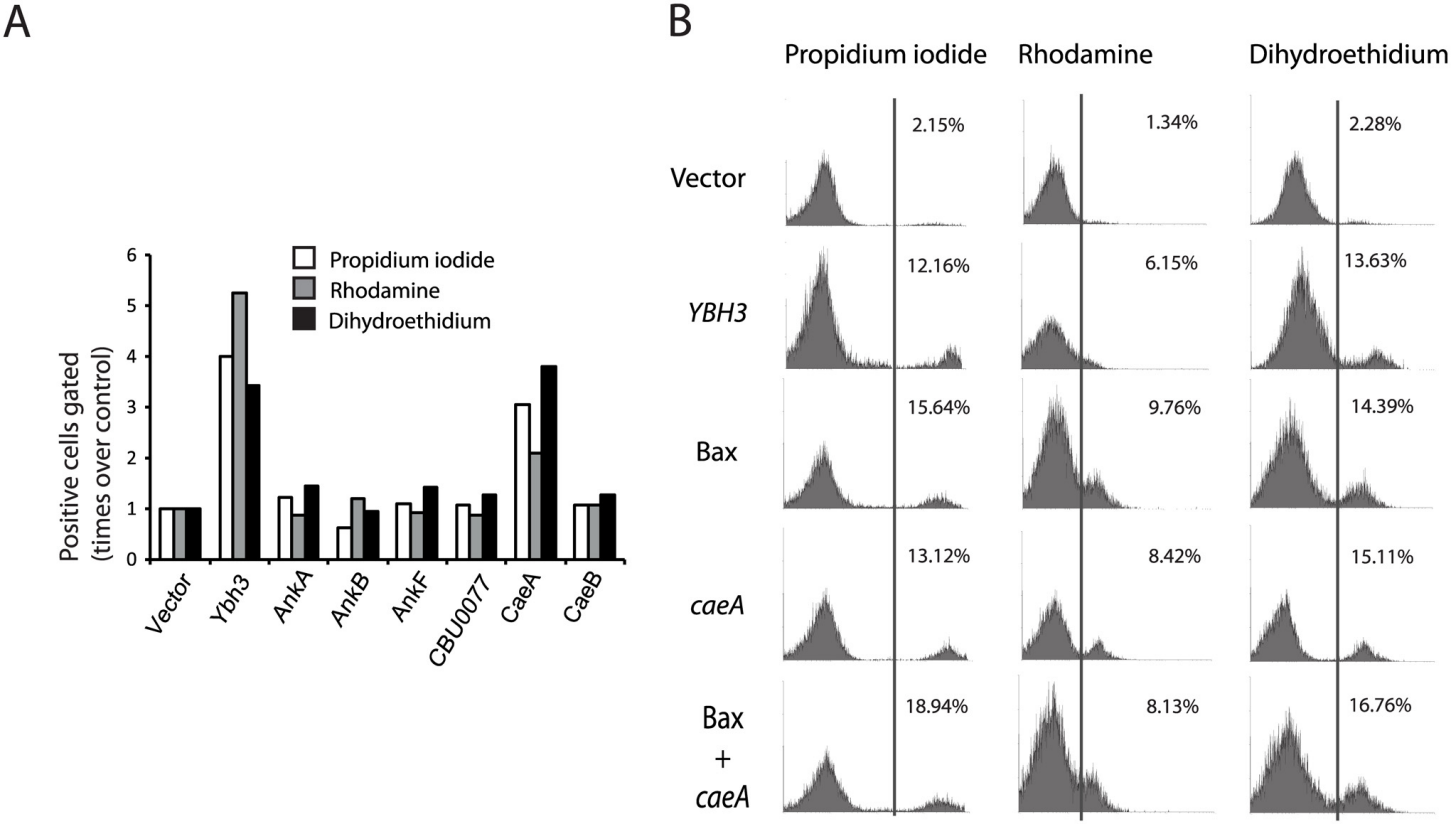
CaeA behaves like apoptotic inducers Ybh3 and human Bax in yeast. (A) CaeA expression induces oxidative stress. The graph represents the enhancement of positive YPH499 yeast cells expressing the indicated *C*. *burnetii* effector proteins from the corresponding *GAL1*-driven expression vectors, as determined by flow cytometry, expressed as times-fold over control cells transformed with the empty vector. The fluorochromes propidium iodide (white bars), rhodamine 123 (grey bars) and dihydroethidium (black bars), respectively monitor cell death, altered mitochondrial potential and intracellular ROS accumulation in the yeast populations. *GAL1*-driven *YBH3* overexpression on the same strain was used as a positive control. Data correspond to a representative experiment of triplicate replicas. (B) Flow cytometry histograms of representative experiments as in (A) on YPH499 transformants expressing the indicated proteins from the corresponding *GAL1*-driven expression vectors. The gating threshold is marked by a vertical line. The percentage of cells in the population beyond the gating threshold is shown for each histogram.

Cae effectors have previously been related to the inhibition of apoptotic pathways in the host cell [15, 17, 27, 28]). In yeast however, CaeA expression was shown to be toxic (Fig 1B and [13, 28]). To determine the molecular mechanism(s) behind this activity, we tested whether loss of selective permeability of the plasma membrane (propidium iodide-positive cells), loss of mitochondrial membrane potential (MMP; rhodamine-positive cells) or production of reactive oxygen species (ROS; dihydroethidium-positive cells), which are markers related to cell death [29], were enhanced or reduced upon expression of *C*. *burnetii* effectors in yeast. Except for CaeA, none of the expressed effectors led to significant alterations in the parameters monitored by these markers, in spite of their toxicity (Fig 3A). Expression of CaeA led to cell death, loss of MMP and production of ROS at similar levels than overproduction of either *YBH3*, a pro-apoptotic *S*. *cerevisiae* BCL-2 Homology domain 3 (BH3)-containing protein [30] or heterologous mammalian Bax [31] (Fig 3B). In contrast, *caeA* expression did not counteract the apoptotic effects induced by either co-expression of Bax (Fig 3B) or acetic acid treatment (S3 Fig). Rather, the deleterious effects caused by CaeA were additive to those of acetic acid, as in the case of Ybh3 and Bax (S3 Fig). Thus, in yeast, unlike in higher cells, *caeA* expression does not protect the cell from apoptotic damage, but leads to oxidative damage in a fashion reminiscent of pro-apoptotic factors.

### CaeA is ubiquitinated and primarily stored at JUNQ compartments in yeast

**Fig 4:**
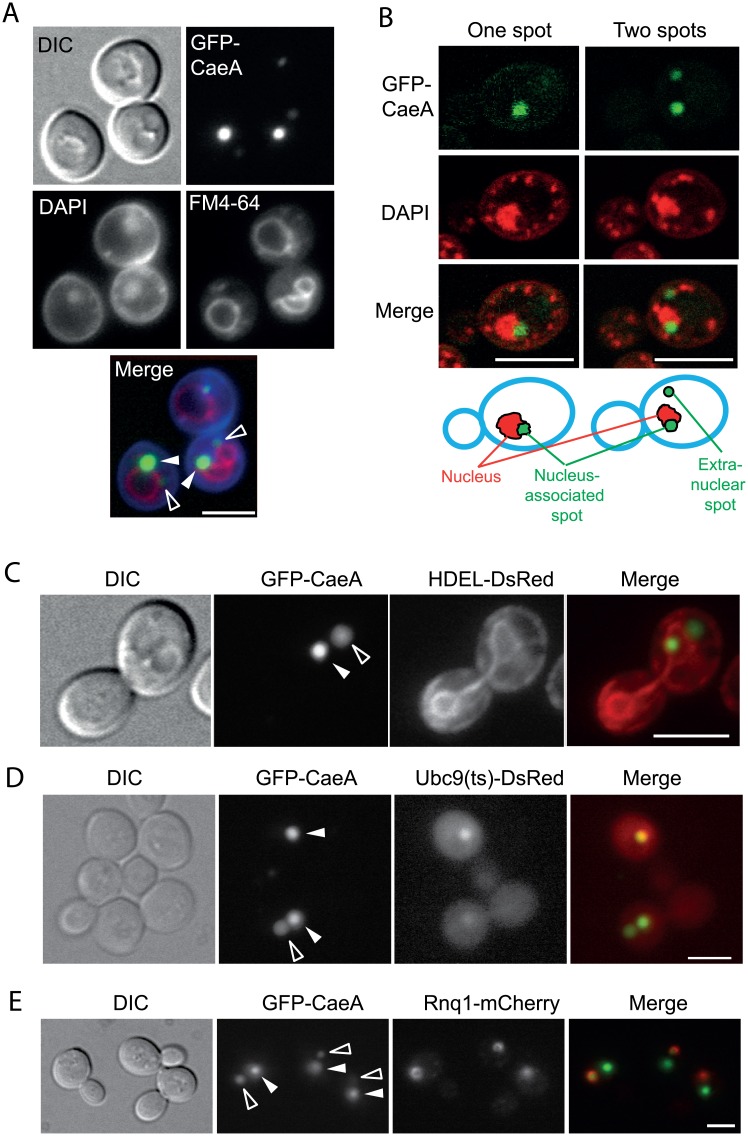
GFP-CaeA primarily accumulates at JUNQs, and residually at IPODs in yeast cells. (A) Fluorescence microscopy of GFP-CaeA-expressing yeast cells counterstained with DAPI and FM4-64 to visualize respectively nuclei and vacuoles. YPH499 cells transformed with pYES2-GFP-CaeA were induced in galactose for 6 h. Filled white arrowheads point to the nucleus-associated spot, normally the most intense; hollow arrowheads indicate the less intense vacuole-associated spot. (B) A prominent GFP-CaeA spot is associated to the nucleus. Confocal microscopy of representative cells displaying one only (left) or two (right) GFP-CaeA spots, as indicated. DAPI, which marks nuclear and mitochondrial DNA, is shown in red color for better contrast with green GFP-CaeA. The relative position of GFP-CaeA spots to nuclear DNA for each cell is depicted in the schemes at the bottom. (C) Representative micrograph of a pYES2-GFP-CaeA transformant of strain VHY87, bearing an integrated marker for the ER (HDEL-DsRed). The filled white arrowhead indicates the GFP-CaeA spot engulfed by the perinuclear ER, and the hollow arrowhead shows the spot located in the cytoplasm beyond the perinuclear ER. (D) Co-localization of GFP-CaeA (green) with the JUNQ marker Ubc9ts-DsRed (red). YPH499 yeast cells were co-transformed with pYES2-GFP-CaeA and pESC-LEU-CHFP-Ubq9ts and induced in galactose for 4 h. The GFP-CaeA positive cell in the upper part of the picture shows a representative cell with one single GFP-CaeA spot, which corresponds to the JUNQ (white filled arrowhead). In cells in which a second, less intense spot appears, it shows no co-staining with Ubc9Ts (hollow arrowhead, bottom cell). (E) Co-localization of GFP-CaeA (green) with the IPOD marker Rnq1 (red). YPH499 yeast cells were co-transformed with pYES2-GFP-CaeA and p425-GAL1-RNQ1-mCherry, and induced as in C. The second, less intense, GFP-CaeA spot is clearly associated to the Rnq1 marker. As in panels (C) and (D), filled white arrowheads point at GFP-CaeA at JUNQs, whereas empty arrows mark IPODs. Bars represent 5 μm in all panels.

Next we investigated whether subcellular localization of heterologous GFP-CaeA in yeast cells could provide clues for its toxicity or the induction of oxidative damage and apoptotic cell death shown above. As shown in Fig 4A, GFP-CaeA localized at one or two spots per cell, rather than in many smaller spots, as observed in a subpopulation of HeLa cells (see Fig 1A). Simultaneous staining with DAPI to visualize the nucleus and FM4-64 as a marker of endocytic pathway suggested that one of the spots was always associated to the nucleus (100% of GFP-positive cells; n = 300), whereas the second spot, usually less intense, was rather associated to the yeast vacuole (100% of cells with two GFP-CaeA spots; n = 300) (Fig 4A). Confocal microscopy of DAPI-stained yeast cells confirmed that the only CaeA-labeled spot, or the most intense when two spots were present, was intimately associated, but never overlapping, with nuclear DNA (Fig 4B). Co-expression of a DsRed fusion to the endoplasmic reticulum (ER) localization signal HDEL [32], which readily marks the perinuclear and peripheric ER, showed that the brightest spot was always (100% of GFP-positive cells, n = 300) engulfed by the perinuclear ER, confirming its intimate association with the nucleus (Fig 4C). The observed GFP-CaeA localization was consistent with the juxtanuclear quality-control (JUNQ), also known as intranuclear quality control (INQ), and the perivacuolar insoluble protein deposit (IPOD) compartments, recently described in yeast and mammalian cells [21, 33]. These compartments were first observed in yeast upon heterologous expression of the von Hippel-Lindau (VHL) tumor suppressor protein or a thermosensitive allele of the SUMO ligase Ubc9 [21]. A feature of these compartments is that they are retained at the mother cell during the budding cycle, so that the newborn daughter does not inherit damaged or defective proteins [34]. Thus, we determined whether medium- and large-budded cells showed fluorescent GFP-CaeA spots either in the daughter or in the mother cell. Out of 300 budding cells observed by fluorescence microscope, 100% displayed GFP fluorescent spots only in the mother cells, whereas not a single bud was observed to host these GFP-CaeA-marked compartments. Furthermore, co-expression of Ubc9ts-DsRed and GFP-CaeA demonstrated that one of the spots, the most conspicuous, corresponded to the JUNQ (Fig 4D). Actually, when only one spot per cell of GFP-CaeA was observed, it always co-localized with Ubc9ts-DsRed, suggesting that the JUNQ is the primary destination of the CaeA protein in yeast. Furthermore, CaeA expression seemed not only to localize but to induce the formation of JUNQs/INQs, as Ubc9ts-DsRed spots could be appreciated in cells incubated at permissive temperature that expressed GST- or GFP-CaeA, but not in those expressing GST or GFP from the empty vectors (data not shown). Moreover, CaeA-(His)_6_, which is not toxic for yeast, did not induce Ubc9ts-DsRed cytoplasmic aggregates either (data not shown), suggesting that a free C-terminal end is required for CaeA to induce protein aggregation. Co-expression of the IPOD marker Rnq1-DsRed demonstrated that the second, less intense, GFP-CaeA spot either overlayed or was surrounded by this marker (Fig 4E). Thus, interestingly, the IPOD seems to accumulate the excess of CaeA when the JUNQ is saturated

**Fig 5:**
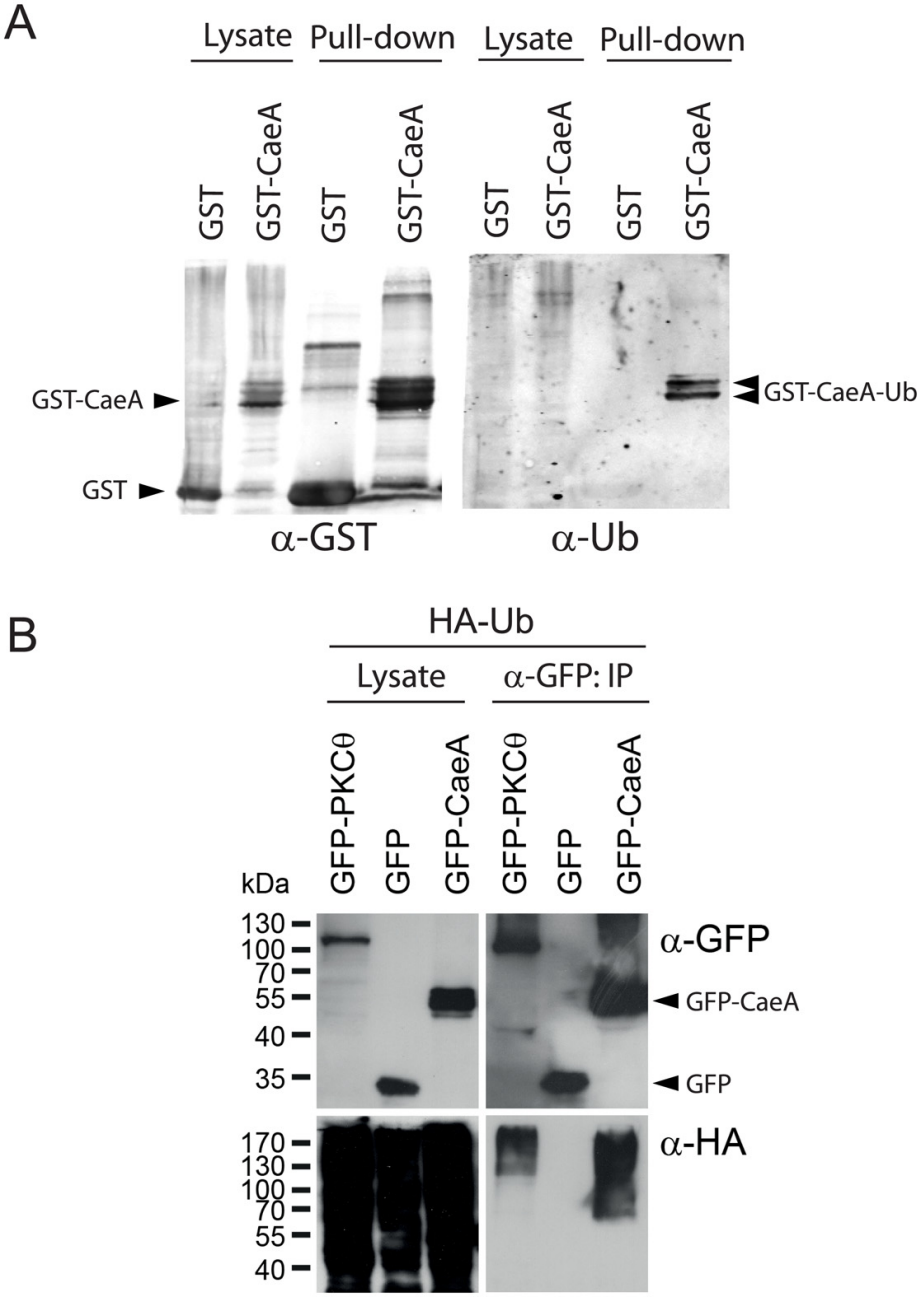
CaeA is ubiquitinated in yeast and mammalian cells. (A) GST-CaeA was affinity purified from YPH499 cell lysates expressing the fusion protein from pEG(KG)-CaeA after 6h-galactose induction. A transformant with the empty plasmid expressing GST alone were analyzed in parallel as a control. Original inputs (Lysate) and the result of affinity purification on glutathione-coated beads (Pull-down) were solved by SDS-PAGE, transferred to a nitrocellulose membrane, and immunoblotted with both anti-GST (left) and anti-ubiquitin (right) antibodies, as indicated. (B) HEK293T cells were co-transfected with plasmids expressing the indicated GFP-fusion proteins and HA-ubiquitin. GFP-tagged proteins were immunoprecipitated (IP) with a rabbit anti-GFP antibody. Lysates and IPs were solved by SDS-PAGE, transferred to a PVDF membrane, and immunoblotted with both anti-GFP (top) and anti-HA antibodies (bottom).

Nucleus-associated JUNQs, but not perivacuolar IPODs, have been reported to store ubiquitinated unfolded proteins [21]. Interestingly, GFP-CaeA showed slow mobility bands in immunoblots on yeast lysates (Fig 2A). To test whether CaeA was ubiquitinated in yeast cells, we expressed GST-CaeA, purified the fusion protein by glutathione affinity chromatography and immunoblotted the pulled down proteins with anti-ubiquitin antibodies. Indeed, the slow mobility bands corresponded to ubiquitinated CaeA forms (Fig 5), likely representing the fraction of protein stored at the JUNQ.

CaeA has a variable series of Glu-Lys (EK) repeats that might account for autoaggregation properties or act as ubiquitination target sequence. We expressed a mutant version lacking the EK repeats (*caeA*Δ49–61) but it behaved as wild type *caeA* in terms of toxicity, post-translational modifications, accumulation in JUNQ/IPOD compartments and triggering MMP depolarization and ROS production (S4 Fig). Thus, this region is dispensable for the effects of CaeA in the yeast cell.

### CaeA is poly-ubiquitinated in mammalian cells

To test whether CaeA is also ubiquitinated in mammalian host cells, we transfected HEK293 cells with GFP-CaeA and HA-ubiquitin and performed a co-immunoprecipitation assay. As a positive control we used GFP-PKCθ and as negative control GFP. As demonstrated in Fig 5B. Both GFP-CaeA and GFP-PKCθ were efficiently poly-ubiquitinated, while GFP was not. This result implies that the ubiquitination of CaeA is conserved in yeast and mammalian cells.

### AnkA accumulates at yeast IPOD compartments

**Fig 6:**
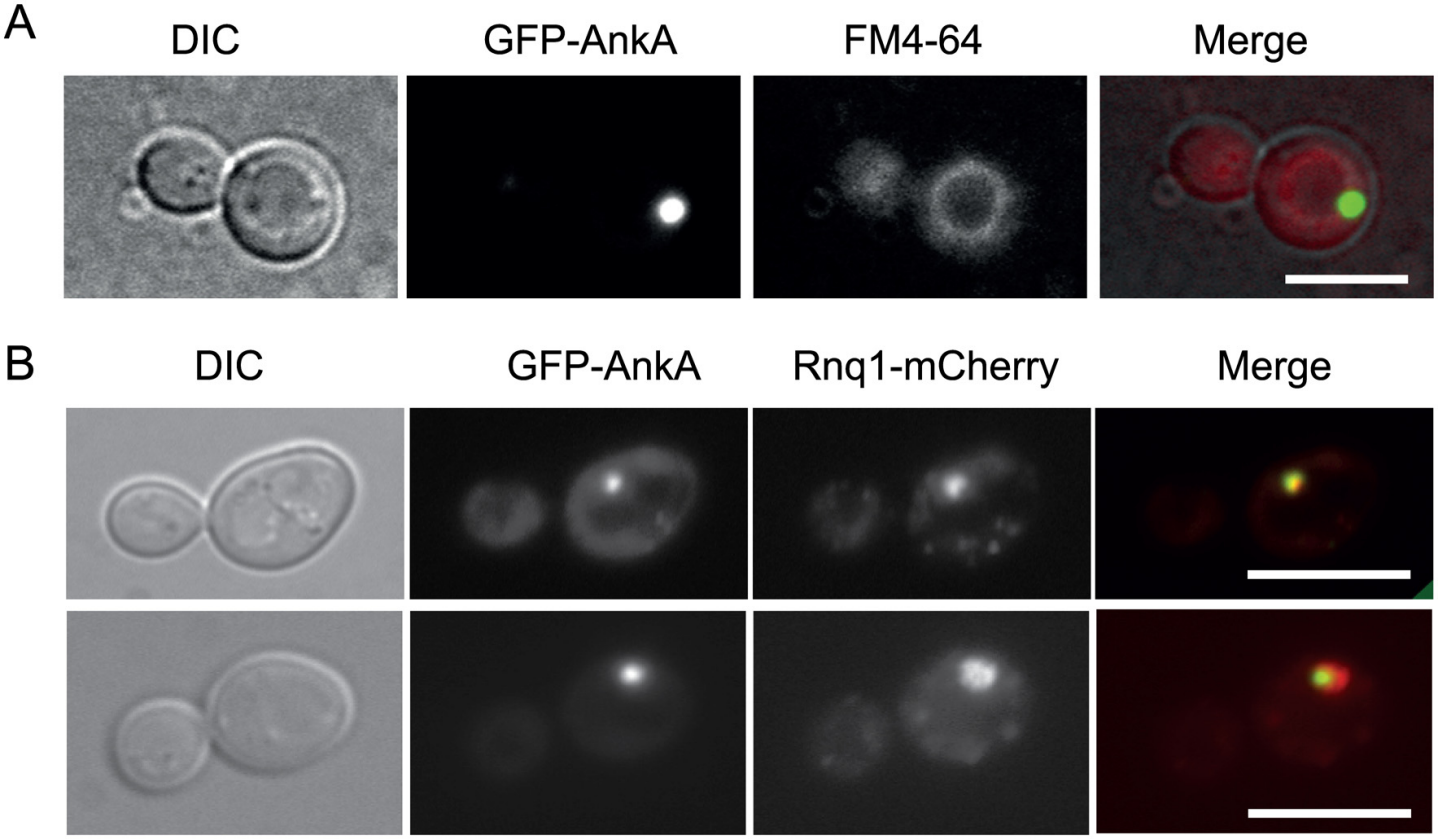
Localization of GFP-AnkA to the yeast IPOD compartment. (A) YPH499 transformants expressing GFP-AnkA (green) from the pYES2-GFP-AnkA plasmid after induction in galactose for 4 h were incubated with FM4-64 (red) for 30 min to allow visualization of the vacuolar membrane. (B) YPH499 yeast cells were co-transformed with pYES2-GFP-AnkA and p425-GAL1-RNQ1-mCherry, an induced as above. Representative cells are shown of two typical patterns: neat co-localization of both red and green channels (upper panel) and inclusion of the green GFP-AnkA signal within a larger Rnq1-mCherry compartment (lower panel). Bars represent 5 μm.

In view of the results obtained with CaeA, we investigated whether other *C*. *burnetii* effectors found to be toxic in yeast were equally targeted to these compartments. No co-localization with JUNQs was observed (data not shown), but GFP-AnkA did accumulate at single large vacuole-associated spots (Fig 6A). These spots were marked by Rnq1-DsRed (Fig 6B), suggesting their nature as IPODs. Thus, GFP-AnkA localizes differently in yeast and mammalian cells, probably due to aggregation and/or misfolding in yeast, as in the case of CaeA. Given that no oxidative or apoptotic markers were detected by expression of AnkA, and its toxicity for the yeast cell was milder than that of CaeA, it is likely that accumulation of unfolded proteins at the JUNQ, but not at the IPOD is related to the high toxicity of CaeA in yeast.

### CBU0077 localizes to the vacuole and abnormal ER membranes in yeast

**Fig 7:**
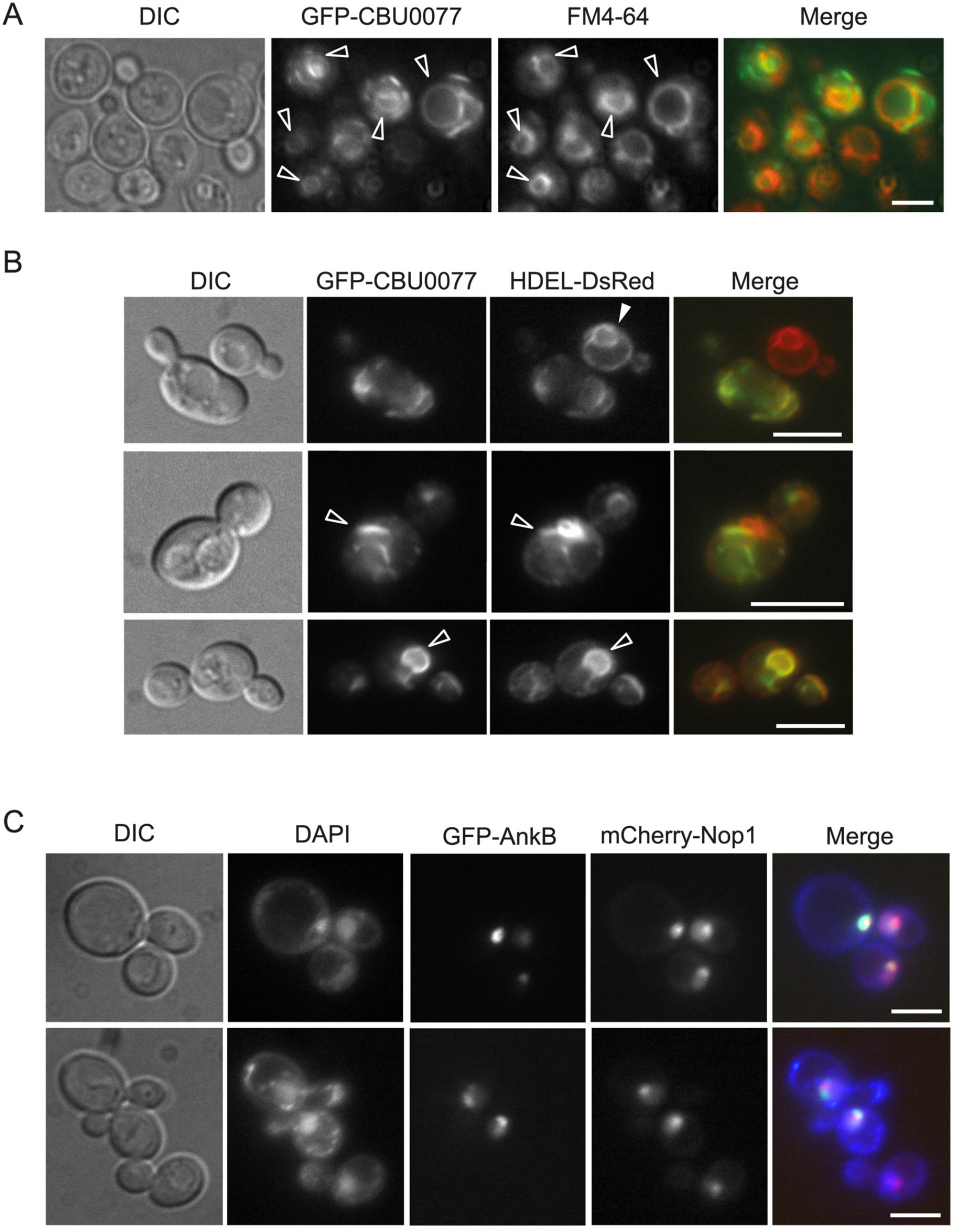
Localization of CBU0077 and AnkB in yeast cells. (A) GFP-CBU0077 is associated to yeast vacuolar membranes. Fluorescence microscopy of YPH499 transformants expressing GFP-CBU0077 (green) from the pYES2-based expression vector and induced with galactose for 4 h prior to incubation with FM4-64 (red) as in Fig 5A. Arrowheads mark vacuoles with membranes clearly marked in both channels. (B) GFP-CBU0077 accumulates at abnormal cytoplasmic structures that harbor the ER marker HDEL. Fluorescence microscopy of pYES2-GFP-CBU0077 transformants of strain VHY87, constitutively expressing the ER marker HDEL-DsRed. The filled arrowhead in the upper panel points to a cell lacking GFP signal, serving as a visual reference for a typical yeast ER shape (red channel) consisting of neat perinuclear and plasma-membrane associated membranes. This pattern is typically distorted in the adjacent cell, showing high GFP-CBU0077 signal. In the middle panel, a characteristic cell in which some of the cytoplasmic bar-shaped cytoplasmic structures containing GFP-CBU0077 (green) co-localize with the ER marker (hollow arrowhead). In the lower panel, the arrowhead indicates a characteristic cell with a high expression level of GFP-CBU0077 strongly associated with the perinuclear ER. (C) Localization of AnkB to the yeast nucleus and association with the nucleolar compartment. YPH499 cells were co-transformed with pYES2-GFP-AnkB and pUN100-mCherry-NOP1, induced in galactose for 4h, co-stained with DAPI to counterstain nuclei and observed by fluorescence microscopy. Representative fields are shown displaying cells at different stages of the mitotic cycle. The GFP-AnkB signal (green) is enriched within the yeast nucleus at a spot either overlapping or adjacent to the mCherry-Nop1 nucleolar marker. Bars correspond to 5 μm in all panels.

As shown above, in mammalian cells localization of CBU0077 depends on the fusion tag. Thus, GFP-CBU0077 decorated mitochondria, whereas FLAG- and HA-tagged CBU0077 co-localized with the lysosomal LAMP-1 marker (Fig 1 and [6]). When expressed in yeast, 76.33 ± 2.31% of the cells displayed GFP-CBU0077 at the vacuolar membrane, an equivalent to lysosomes in mammalian cells, as shown by staining with the endocytic marker FM4-64 (Fig 7A). However, other cytoplasmic structures, usually in the shape of bars that were not stained by FM4-64, were also labeled with GFP-CBU0077 (Fig 7A). Co-expression with HDEL-DsRed probe revealed that these structures corresponded to ER membranes. A very high proportion (95.66 ± 1.53%) of cells with GFP-CBU0077 signal showed alterations in the ER shape and organization (see representative cells in Fig 7B), as compared with GFP-negative cells (marked with a white arrow in the upper panel of Fig 7B), which display the normal ER morphology reported by using this probe [32]. Such alterations involved the lack of a homogeneous perinuclear and plasma membrane-associated ER, the appearance of cytoplasmic bars and disorganized structures, and enhancement of perinuclear signal. Thus, interestingly, CBU0077 expression co-localizes with and distorts the yeast ER.

### AnkB localizes to the yeast nucleus, and is associated to the nucleolus

Similar to its localization when ectopically expressed in HeLa cells, GFP-AnkB localized to the yeast nucleus, as confirmed by DAPI staining (Fig 7C). Moreover, GFP-AnkB signal was enriched at a particular kidney-shaped spot within the nucleus. Co-expression of a marker for the yeast nucleolus, Nop1-mCherry, indicated that GFP-AnkB invariably concentrated at the nucleolus or in close proximity to it (Fig 7C). Remarkably, nuclear localization of AnkB in yeast is consistent with that observed when ectopically expressed in HeLa cells (see Fig 1A), strongly suggesting that AnkB undergoes nuclear translocation when produced in eukaryotic cells.

### AnkB alters yeast sensitivity to aminoglycosides

**Fig 8:**
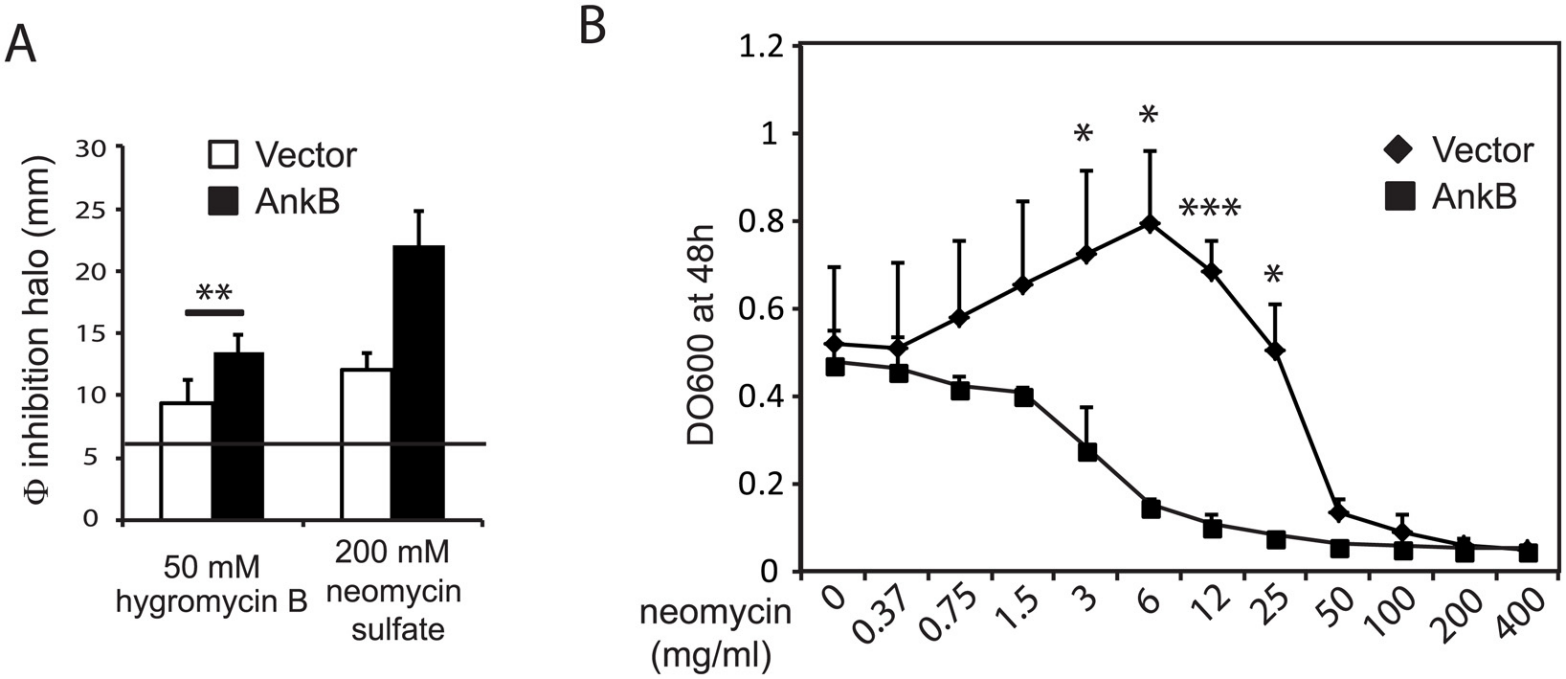
AnkB expression enhances *S*. *cerevisiae* sensitivity to aminoglycosides. (A) Graph displaying the measurements of the growth inhibition halo (mm) around a 6 mm-disc (horizontal line) soaked with a solution of the hygromycin B or neomycin sulfate concentrations indicated on YPH499 cells bearing pYES2-GFP-AnkB or the empty vector as a control. Data are the average of five (hygromycin B) or two (neomycin) independent experiments performed on different transformants. (B) Dose-response curve showing the effect of ½ serial dilutions of neomycin on growth of YPH499 transformants bearing pYES2-GFP-AnkB or the empty vector, as a control, cultured in SCGal liquid medium. Data are the average or three biological replicates, each performed on an individual transformant clone. Asterisks denote statistical significance of data (*) p<0.05; (**) p<0.01; (***) p<0.005. P-values were calculated by the Student’s T-test.

Next we studied whether heterologous expression of *C*. *burnetii* effectors caused cellular stress in the yeast cell. A study on the activation of yeast stress-induced MAPK signaling pathways related to mammalian Erk- and p38-mediated routes was performed by immunoblotting yeast lysates with two antibodies that specifically recognize the activated dually phosphorylated form of either MAPKs Slt2, Fus3 and Kss1 or the MAPK Hog1. No evidence of interference of any of the *C*. *burnetii* proteins with these pathways was found (data not shown). To gain insight into the mechanisms leading to toxicity in yeast, we exposed cells expressing *C*. *burnetii* effectors to a battery of compounds targeting different processes in the cell, namely the antifungals amphotericin B and miconazole, the cytoskeleton polymerization inhibitors latrunculin and benomyl, TORC inhibitor rapamycin, myriocin, tunicamycin, menadione and aminoglycoside antibiotics, by a halo sensitivity assay on solid medium. As a result, we found that expression of GFP-AnkB, but not the other effectors, affected yeast sensitivity to aminoglycosides, like neomycin sulfate and hygromycin B (Fig 8A). Sensitivity of AnkB-expressing cells to neomycin was further confirmed by a dose-response curve (Fig 8B).

## Discussion

The difficulties of working with obligate parasitic bacteria such as *C*. *burnetii* in mammalian cells make the development of model systems necessary. The yeast *S*. *cerevisiae* has been used to study virulence factors from different bacterial pathogens and, very recently, it has been applied to the characterization of several type IVB secretion system effectors from *C*. *burnetii* [6, 12, 13]. Here we exploit this model as a tool to study six particular effector proteins from this bacterium. In this context, the expression of most *C*. *burnetii* effectors tested inhibited yeast growth to a different extent, including CaeA, which has been described as a T4BSS pro-survival effector in higher cells [17]. This controversial finding was consistent with previous reports [6, 13], so we focused on understanding the effects of CaeA on yeast cells. Paradoxically, CaeA in yeast rather behaved as a pro-apoptotic effector, altering mitochondrial membrane potential, increasing cell lysis, promoting generation of ROS, and exacerbating the effect of pro-apoptotic agents or proteins in yeast. Furthermore, we observed that GFP-CaeA was partially ubiquitinated. Interestingly, this result was extrapolated to higher cells, where ectopically expressed GFP-CaeA was also found to undergo ubiquitination. It remains to be elucidated whether this post-transcriptional modification might be important for function. Our results suggest that ubiquitinated CaeA is stored at JUNQ/INQ in yeast cells, in agreement with the notion that misfolded proteins that accumulate at the JUNQ are generally ubiquitinated, whereas those destined to the IPOD are not [21]. Therefore, the partial ubiquitination of CaeA is consistent with its localization in both types of compartments, although it seems to preferentially accumulate at JUNQs. Such localization of CaeA is likely due to a folding defect in the heterologous system. The sustained proteostatic stress generated by the overexpression of a foreign protein might account for the observed enhancement in oxidative stress markers and general toxicity of CaeA on the yeast cell. However, not all of the *C*. *burnetii* effectors causing toxicity in yeast end up in protein quality control compartments. Among the T4BSS substrates other than CaeA assayed here, only AnkA accumulated in IPODs, underscoring the idea that extra toxicity of CaeA could be related to its specific accumulation at the JUNQ.

Although our results in yeast likely reflect a consequence of overexpression of the heterologous protein, the fact that CaeA partially accumulates in discrete punctate spots in mammalian cells might indicate that ectopically expressed CaeA could also be destined to yet undescribed protein quality control compartments. Peculiarly, only a very limited number of overproduced proteins have been reported to display this behaviour in yeast, so it might reflect structural properties intrinsic to CaeA. In consequence, CaeA could be potentially utilized as a tool to study protein quality control and ubiquitin-dependent sorting pathways in yeast.

In mammalian cells, some differences in the localization of ectopically expressed effectors were observed that differ with previous reports. For example, AnkA and AnkB were shown by Voth *et al*. [35] to be cytoplasmic. Here, GFP-AnkA is indeed found at the cytoplasm, although in a reticular pattern, partially associated with tubulin. AnkB, however, is neatly concentrated in the nucleus, although we cannot discard that this localization does not correspond to the full-length protein, but to a major C-terminal truncated form that may result from proteolysis, as suggested by immunoblot analysis. Divergence with previous reports may be due to the experimental choice of different fusions and *C*. *burnetii* strains. CaeB was previously reported to be associated to a mitochondrial fraction [6], although its localization in a later report was suggestive of wider cytoplasmic membrane networks [17], in consistence with the co-localization with the ER calnexin marker here shown.

CBU0077 is a translocated *C*. *burnetii* effector of yet unknown function [36], which had been previously reported to interfere with yeast growth [6]. Inside the host cell, *C*. *burnetii* survives and replicates within compartments named *C*. *burnetii*-containing vacuoles (CCV). The CCV fuses with vesicles bearing early and late endosomal, as well as lysosomal markers. Eventually, the CCV enlarges drastically, even occupying almost the entire volume of the host cell [37]. The formation of the large mature CCV requires interaction with ER membranes through the early secretory pathway [38]. The modulation of such trafficking events is probably orchestrated by *C*. *burnetii* effectors, as bacterial protein production is required for the development of the large CCV [39]. Addressing the localization of *C*. *burnetii* effectors in the yeast model should provide preliminary clues on their putative interaction with eukaryotic intracellular membranes. The localization of CBU0077 at the yeast vacuolar membrane, equivalent to lysosomes in higher cells, as well as at ER membranes, together with the observation that it dramatically alters the morphology of the yeast ER, might suggest that this effector could be implicated in CCV formation or maintenance. However, it remains to be determined where CBU0077 localizes once translocated by *C*. *burnetii* during infection, as ectopically expressed CBU0077 in HeLa cells displayed divergent subcellular localization in lysosomes or mitochondria depending on the fusion-tag.

The ankyrin repeat-containing protein AnkB, a T4BSS effector of elusive function, localized to the nucleus in yeast and mammalian cells. In yeast, AnkB mainly concentrated at the nucleolus. Thus, it is likely that it exerts its toxicity on the yeast cell by modulating gene expression or genome stability. There is limited evidence of bacterial effectors that translocate to the nucleus and directly modulate nuclear functions in the host cell. Interestingly, such effectors often seem to contain ankyrin repeats, such as AnkA from *Anaplasma phagocytophilum* [40, 41]. Yeast cells overexpressing AnkB were more sensitive to aminoglucosides, which might hint that its interference with nuclear functions negatively affects protein synthesis, perhaps by altering proper expression of ribosomal genes. In a screen for overexpression suppressors, we isolated the *GCV3* gene, coding for the H-subunit of the yeast mitochondrial glycine decarboxylase complex [42], by its ability to counteract AnkB-induced growth inhibition (data not shown). Thus, it is likely that the presence of AnkB at the nucleus perturbs metabolic or mitochondrial function. Further studies will be required to elucidate which particular functions are downregulated by this nuclear translocated effector and whether its nuclear targets are common to yeast and higher cells.

In sum, heterologous expression in yeast as an approach to understand the interaction of bacterial translocated effectors with eukaryotic cellular functions may prove helpful [7, 43], especially when novel effectors are under study. Subcellular localization of heterologous bacterial effectors might provide important clues to preliminarily understand the organelles, pathways and functions targeted by these proteins. However, it must be considered that yeast, as a heterologous model, has severe limitations. Therefore, caution should be taken in the interpretation of results and their extrapolation to higher cells. Here we underscore that some bacterial effectors accumulate in protein quality control compartments, such as JUNQs or IPODs, a phenomenon that could reflect misfolding or aggregation of the overexpressed protein. Thus, in these cases, proteostatic stress may significantly contribute to the observed inhibition of yeast growth, which would not be necessarily related to interference with molecular targets conserved from yeast to human. In turn, these aggregative proteins might shed light on the yet obscure mechanisms that govern protein quality control in eukaryotic cells. On the other hand, in proteins neatly targeting conserved cellular compartments, such as trafficking organelles or the nucleus, as it is the case of *C*. *burnetii* CBU0077 and AnkB, respectively, yeast might provide useful information on how to orient further analyses in more complex experimental infection models.

## Supporting Information

S1 Fig GFP-AnkA partially co-localizes with tubulin in HeLa cells. A representative confocal image of a HeLa cell transiently expressing GFP-AnkA, stained with an anti-tubulin antibody. Co-localization, as indicated by shades of yellow, was analyzed in four different regions (1 to 4) of a representative image (scale bar, 10 μm).
(EPS)

S2 Fig Growth of yeast expressing GFP-/GST-fusions of *C*. *burnetii* effectors at both 28 and 37°C. Serial dilution drop assays to monitor growth under induction (Galactose) and control (Glucose) conditions of yeast cells expressing the indicated proteins incubated for three days at 28 or 37°C.
(EPS)

S3 Fig CaeA expression enhances severity of apoptotic stimuli. Flow cytometry histograms of representative experiments on YPH499 transformants overproducing from *GAL1*-driven expression pYES2-GFP-based vectors the proteins indicated, after treatment with 125mM acetic acid. The fluorochromes tested, propidium iodide, rhodamine 123 and dihydroethidium, respectively cell death, altered mitochondrial potential and intracellular ROS accumulation indicated in the population, as indicated on top of each column. The gating threshold is marked by a vertical line. The percentage of cells in the population beyond the threshold is shown.
(EPS)

S4 Fig Deletion of the Lys-Glu repetitions of CaeA does not influence the phenotypes induced in yeast. (A) Immunoblot with anti-GFP antibodies on yeast lysates obtained under induction conditions showing expression of GFP fusions of the proteins indicated. Transformant clones of YPH499 strain were grown on SCRaf selective medium and induced for 4 h by addition of galactose. (B) Serial dilution drop assays to monitor growth under induction (Galactose) and control (Glucose) conditions of representative transformant clones expressing the indicated proteins. (C) GFP-CaeA-Δ49–61 is located at the proximity of the nuclear and vacuolar compartments, in a fashion identical to wt CaeA. (D) Flow cytometry histograms of representative experiments on YPH499 transformants overproducing from *GAL1*-driven expression vectors the proteins indicated in presence or absence of 125mM acetic acid. The fluorochromes tested, propidium iodide, rhodamine 123 and dihydroethidium are indicated. The gating threshold is marked by a vertical line. The percentage of cells in the population over the threshold is shown.
(EPS)

S1 Table Summary of observed localization and effects of ectopically expressed *C*. *burnetii* effector fusions. (DOCX)
