# Supplementary material for: Studying Coxiella burnetii Type IV Substrates in the Yeast Saccharomyces cerevisiae: Focus on Subcellular Localization and Protein Aggregation
Source: PLoS One. 2016 Jan 28;11(1):e0148032. doi: 10.1371/journal.pone.0148032 (PMC4731203; doi:10.1371/journal.pone.0148032)
Supplement: S1 Table — (DOCX) [file pone.0148032.s005.docx]

**S1 Table**. **Summary of observed localization and effects of ectopically expressed *C. burnetii* effector fusions**.

| ***C. burnetii* effector fusion** | **Localization in cell lines** | **Localization in yeast** | **Toxicity in yeast** | **Additional phenotypes in yeast** |
| --- | --- | --- | --- | --- |
| **GFP-AnkA** | Partial co-localization with tubulin | IPODs | + | - |
| **GST-AnkA** | ND | ND | + | ND |
| **GFP-AnkB** | Nucleus | Nucleus | + | Sensitiveness to hygromycin B and neomycin |
| **GST-AnkB** | ND | Nucleus | + | Sensitiveness to hygromycin B and neomycin |
| **GFP-AnkF** | Cytoplasm and nucleus | Cytoplasmic spots | - | - |
| **GST-AnkF** | ND | ND | - | ND |
| **GFP-CBU0077** | Mitochondria | ER and vacuolar membrane | + | - |
| **CBU0077-(His)_6_** | ND | ND | - | ND |
| **GFP-CaeA** | Diffuse nuclear or Multiple bright punctate dots (40%) | JUNQs/INQs  and  IPODs | ++ | Enhanced ROS production and loss of MMP |
| **GST-CaeA** | ND | ND | + | Enhanced ROS production and loss of MMP |
| **CaeA-(His)_6_** | ND | - | - | - |
| **GFP-CaeB** | ER | Cytoplasmic spots | - | - |

ND: Not determined.
